# Laplacian of Gaussian for Fast Cell Detection and Segmentation in Cervical Cytology to Help in Cancer Diagnosis

**DOI:** 10.7759/cureus.78519

**Published:** 2025-02-04

**Authors:** Jesus E Alcaraz-Chavez, Adriana C Téllez-Anguiano, Juan C Olivares-Rojas, Gerardo M Chávez-Campos

**Affiliations:** 1 Graduate Studies and Research Division, TecNM Instituto Tecnológico de Morelia, Morelia, MEX

**Keywords:** cancer diagnosis, cervical cytology, image processing and analysis, lapacian of gaussian, segmentation

## Abstract

Cervical cancer remains one of the leading causes of mortality among women worldwide, and its early detection is crucial to improve survival rates. While a Pap smear is widely used as a diagnostic tool, it has limitations in sensitivity and specificity due to the inherent subjectivity of cytological analysis. This study proposes a methodology for cervical cell segmentation and extraction based on the Laplacian of Gaussian (LoG) algorithm, which enables the generation of regions of interest to detect and segment cells precisely in cervical cytology samples. Over 2,000 digital images of Pap smear slides were analyzed, derived from 500 cervical cytology slides provided by the State Public Health Laboratory of Michoacán, México. The dataset results demonstrated an accuracy of 96.5%, a recall rate of 99.2%, and an F-measure of 97.8%. Furthermore, the methodology was optimized for real-time analysis, allowing efficient segmentation and detection of cells and their morphological variations. This methodology not only significantly improves accuracy and efficiency in cervical cell segmentation but also has a high potential for application in other experiments that require precise cell segmentation despite morphological variations. In this regard, it offers an adaptable and versatile approach, making a substantial contribution to cytological studies and establishing itself as an effective process to extract cervical cells automatically in real time.

## Introduction

Cervical cancer represents one of the leading threats to women’s health globally. With over one million estimated cases worldwide, many patients do not receive early diagnosis or timely access to treatment [1]. Rapid population growth, aging populations, and the epidemiological transition have contributed to the global increase in cases. According to the World Health Organization (WHO), cervical cancer is the fourth most common cancer among women, with 604,000 new cases and 342,000 deaths in 2020, predominantly affecting low- and middle-income countries [2,3].

While a Pap smear is the standard screening method, its effectiveness depends on the quality of the healthcare system and rigorous control of diagnostic processes. The interpretation of results is subjective, depending on the cytotechnologist’s experience, which can lead to diagnostic errors. Manual analysis, still prevalent in many health centers, is subject to errors caused by the visual fatigue of cytotechnologists. 

Analysts must understand nuclear morphology and the relative dimensions of cellular components. Early pioneers in cervical cytology laid a solid foundation by meticulously studying the differences between benign and neoplastic processes through careful measurements in conventional cytology [4,5]. Although contemporary literature is limited in considering measurements on liquid-based preparations, size relationships remain a key factor in defining diagnostic entities and functional states.

Cervical cytology is primarily used to detect squamous cell carcinoma of the cervix and its precursor lesions. Cell detection in a microscopic field represents significant challenges, especially in the context of the Pap smear, where samples often do not meet optimal staining standards nor provide adequate cellular representation for detailed analysis. This issue complicates the identification of multiple cells within a single visual field. However, due to the wide range of reactive cytomorphological changes, diagnostic criteria require greater precision, and the reproducibility of the results is often limited [6-10]. The detection process must effectively adjust to variations in staining quality and sample conditions in cytological screening. This adaptability is crucial since, even in suboptimal samples, it is possible to identify cancerous or precancerous cells.

Even though consolidated datasets exist in scientific literature, the inherent variability and preprocessing of the samples differ significantly from real conditions observed in cervical cytology. This highlights the importance of adapting and validating diagnostic methods in real clinical contexts. In this context, and due to the inherent limitations of the Pap smear method, there is an urgent need for accurate cell extraction, whether for morphological specialization or the efficient generation of datasets. Scientific literature highlights the importance of having well-defined cellular data, as detecting cells in a general microscopic field is a considerable challenge.

Given the inherent limitations of the current method, several technologies have been proposed to improve the efficiency and accuracy of cervical cancer detection while reducing the subjectivity of the process [11-15].

Generally, the algorithms used for cervical cancer detection are divided into two stages: cytoplasm and nucleus segmentation, followed by detection and classification. For instance, Zhao et al. addressed the low accuracy of traditional methods in the segmentation of adherent cervical cells [16]. In their study, the authors propose an innovative convolutional neural network based on star-convex polygons, called SPCNet, which follows an encoder-decoder structure. 

Similarly, Win et al. used an iterative shape-based method for detecting cell nuclei, employing marker-controlled watershed techniques for separating overlapping cytoplasms and integrating image segmentation, feature extraction, and selection techniques [17]. 

Lindeberg [18] demonstrated automatic feature detection at scale. This work proposes interesting scale levels in image data, considering that local extrema across scales of different combinations of y-normalized derivatives are likely candidates to correspond to relevant structures. Specifically, it shows how this idea can be used as an important mechanism in algorithms for automatic scale selection, adapting the local processing scales to the local image structure. 

Marsh et al. [19] used the Hessian method for precise detection in atomic force microscopy images. Di Ruberto et al. [20] proposed a novel method using the Laplacian of Gaussian (LoG) to recognize white blood cells in microscopic blood images and classify them as healthy or affected by leukemia. 

Xu et al. [21] proposed a joint blob detector based on U-Net, a deep learning model, and Hessian analysis to overcome these issues and identify precise blobs in noisy medical images. Similarly, Gang Wang et al. [22] proposed an automated method to detect overlapping bloblike objects in images, using an iterative LoG filtering approach to reduce overlapping areas of adjacent objects while preserving isolated ones. 

Chen and Zhang [23] presented a two-stage framework based on the mask RCNN. Similarly, some of these approaches rely on a dataset provided by Phoulady and Mouton [24], a cervical cytology dataset. In addition, Wang et al. [25] proposed a segmentation algorithm that enhances the radial contour of nuclei to address cell overlap in cervical cytology images. 

Hoque et al. [26] utilized adaptive thresholding and convolutional filtering for cell segmentation. Similarly, Chowdary et al. [27] proposed three models: one based on the residual-squeeze-and-excitation module, a feature extraction model based on fusion, and a classification model using a multilayer perceptron. All these models aim to segment cells in cervical cytology accurately. 

Furthermore, Hao et al. [28] proposed an improved method to segment the nucleus and cytoplasm in cervical cells based on a deep convolutional network. S and Panicker [29] proposed a deep-learning model called EfficientDet for image segmentation through rigorous analysis of modern detection prototypes. They discovered methods to improve computational efficiency, leading to the creation of EfficientDet. 

Pallavi et al. [30] presented an artificial intelligence-assisted tool to detect cervical dysplasia from cytology images. It uses a pixel-based segmentation approach and two-stage cell classification. The random forest (RF) classifier achieved 99.07% accuracy in cell segmentation, while the artificial neural network (ANN) reached 97.5% accuracy in detecting cervical dysplasia. 

Although the approaches mentioned focus on segmentation through various deep-learning models, the methodology proposed in this article is focused on cell detection based on the detection of regions of interest (blobs) using the LoG with a normalized scale. The dataset used was provided by the State Public Health Laboratory of Michoacán (LESPM), and the cervical cytology images were not preprocessed before applying blob detection analysis. It is important to note that the proposed approach is versatile and can be applied to static images and cervical cytology videos. 

This methodology was applied in previous work to create a knowledge base using scale-invariant feature transform (SIFT) descriptors. It allowed the identification of cell types in cervical cytology without resorting to exhaustive machine learning or deep learning strategies. 

Different configurations of microscopes and mobile devices were implemented, allowing for real-time cell extraction, either through direct connection of the device to the microscope or through the analysis of videos or images of cervical cytologies. Thus, this work does not specify the models of microscopes, cameras, or the technical parameters used. This is a deliberate approach aimed at ensuring the adaptability of the proposed method to various levels of technological equipment in laboratories, including those with limited access to advanced technology.

## Materials and methods

In this work, the developed algorithm aims to improve adaptability to non-ideal smears and image capture devices with different resolutions to analyze cervical cytology using Pap smears. TecNM Instituto Tecnológico de Morelia Ethics Committee issued approval (no. 099.1).

**Figure 1:**
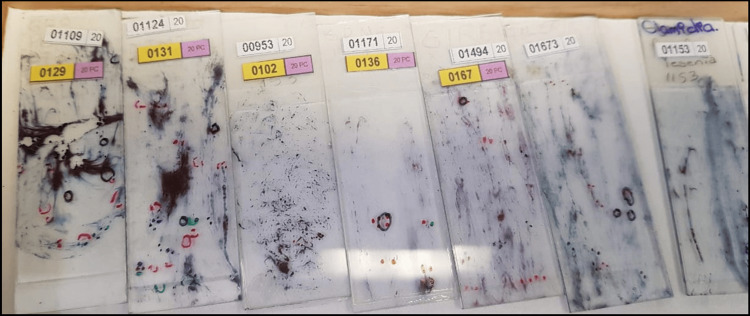
Slides provided by the State Public Health Laboratory of Michoacán (SPHLM) showing samples with varying levels of staining and diverse diagnoses.

In this context, the proposed methodology is based on a wide range of samples provided by the State Public Health Laboratory of Michoacán, Mexico, as illustrated in Figure 1. 

The detection process must effectively adjust to variations in staining quality and sample conditions in cytological screening. This adaptability is crucial since, even in suboptimal samples, it is possible to identify cancerous or precancerous cells. Furthermore, the speed at which specialists examine samples under the microscope has driven the implementation of a real-time video system to optimize the efficiency of the evaluation process. 

This study does not specify a particular type of microscope or camera, as it is not focused on specific equipment, exclusive parameters, or configurations. The methodology aims at the inclusion of laboratories with varying levels of technological resources, acknowledging that not all facilities have access to cutting-edge equipment.

**Figure 2:**
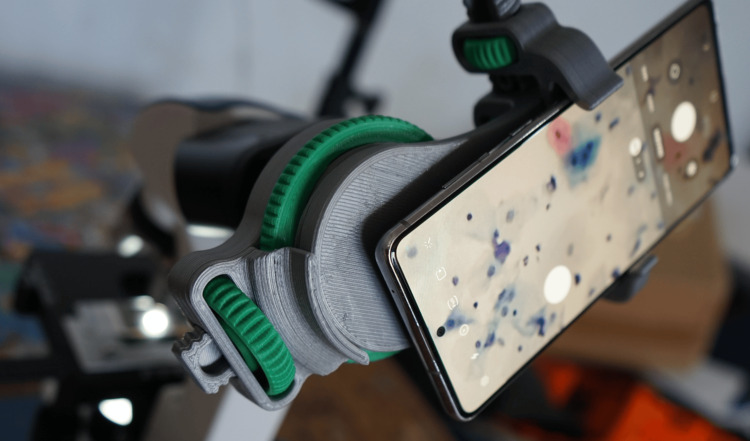
Images and videos captured using the microscope adapter.

To improve the image's capture, a microscope adapter was implemented, and it was fabricated using 3D printing techniques. This device significantly enhanced the functionality of mobile device cameras, optimizing image acquisition and video recording during the study. The adapter is shown in Figure 2.

Using this tool, over 15,000 images and 20 hours of video of cytological specimens were acquired, thoroughly documenting the microscopic fields. It is important to note that the focal distance and magnification level in the microscopic field vary with each observation. Therefore, the quality of cervical cytology images is influenced by several factors, such as the type of mobile device used, focal distance, lens, objective, lighting, and the filter applied to the microscope.

**Figure 3:**
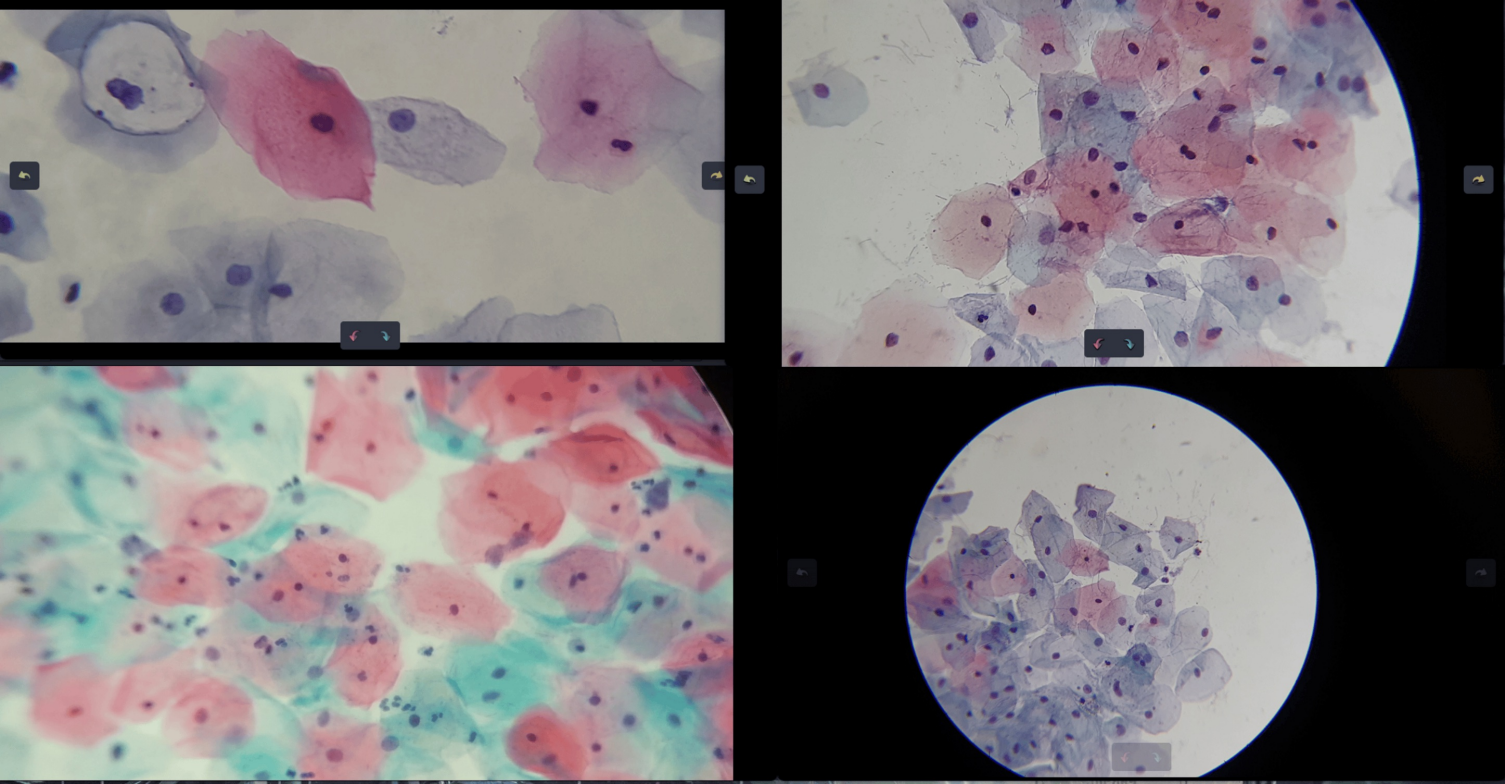
Fields of cervical cytology captured at various focal distances and with different levels of zoom

Despite these variations, the developed methodology allows for the precise detection of individual cells, as long as they are distinguishable within the microscopic field. Figure 3 shows cervical cytology images captured at different focal distances and with varying levels of zoom.

In this context, the normalized scale LoG algorithm was used to segment and extract the different types of cells present in the cytological samples, utilizing the generated regions of interest and accounting for staining variations. The main goal is to contribute to the identification of precancerous lesions, providing a more precise cytological analysis that enables a clear differentiation between cervical cytology samples positive and negative for cancer.

However, this study focuses on detecting and extracting all visible cells in cervical cytology samples using the described technique, limiting its scope to this specific type of segmentation. In addition, the developed method intends to be applicable for real-time cytology (Pap smear) analysis using mobile devices connected to microscopes, to ease detection in clinical settings.

Cervix cytology

Cervical cytology, also known as cervicovaginal cytology or Pap smear, analyzes exfoliated cells from the squamocolumnar junction of the cervix. For years, it has been the primary method for cervical cancer detection and is widely recognized by cancer control and prevention programs for its contribution to reducing both the incidence and mortality associated with this disease [31].

**Figure 4:**
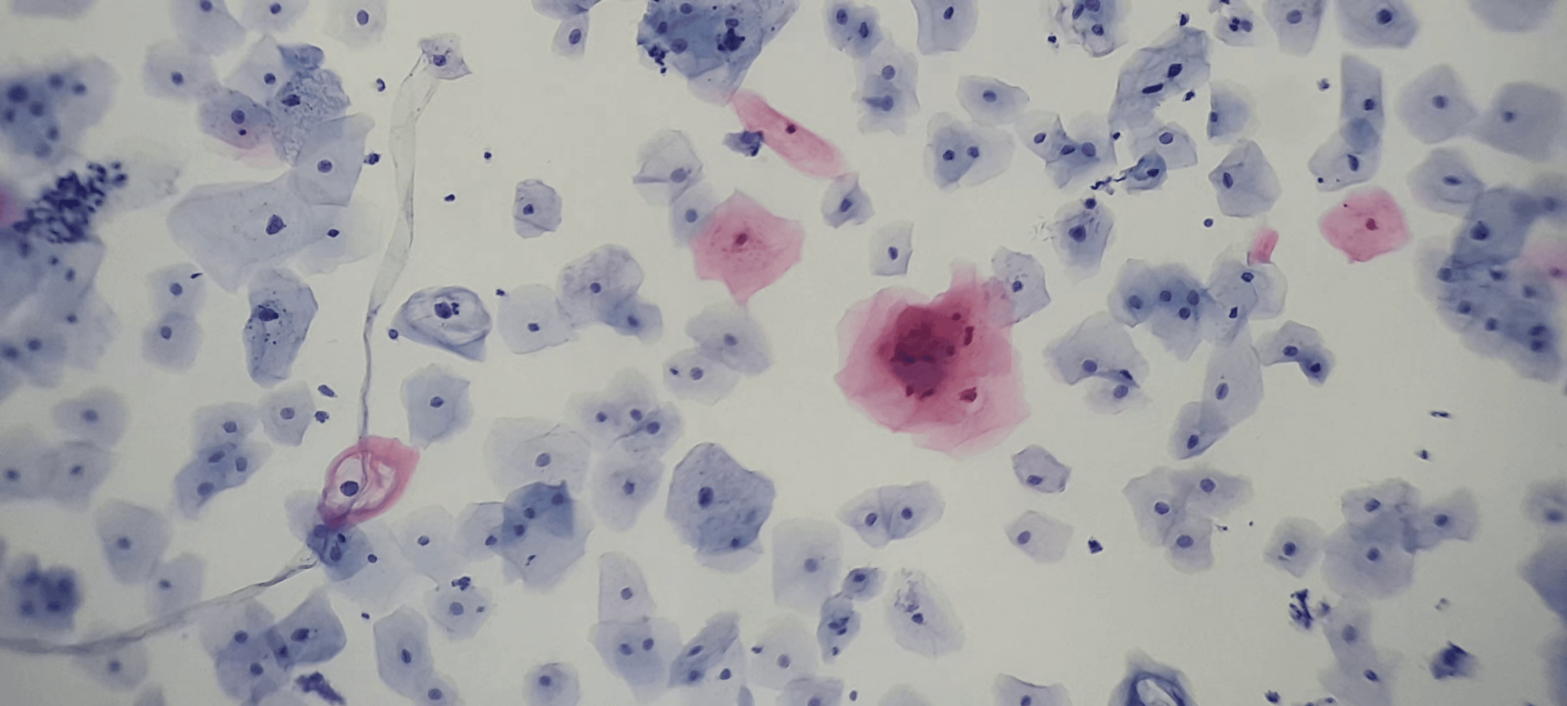
Low-grade cervical cytology lesion, taken with 5x zoom on a mobile device and a 10x microscope objective

Early detection of cervical cancer, before symptoms appear, can significantly increase the chances of a full recovery. This test is often complemented by detecting the human papillomavirus (HPV), one of the main factors associated with this type of cancer. Figure 4 shows cells from a cervical cytology sample, each with specific characteristics of shape, coloration, and nuclear size [32].

**Figure 5:**
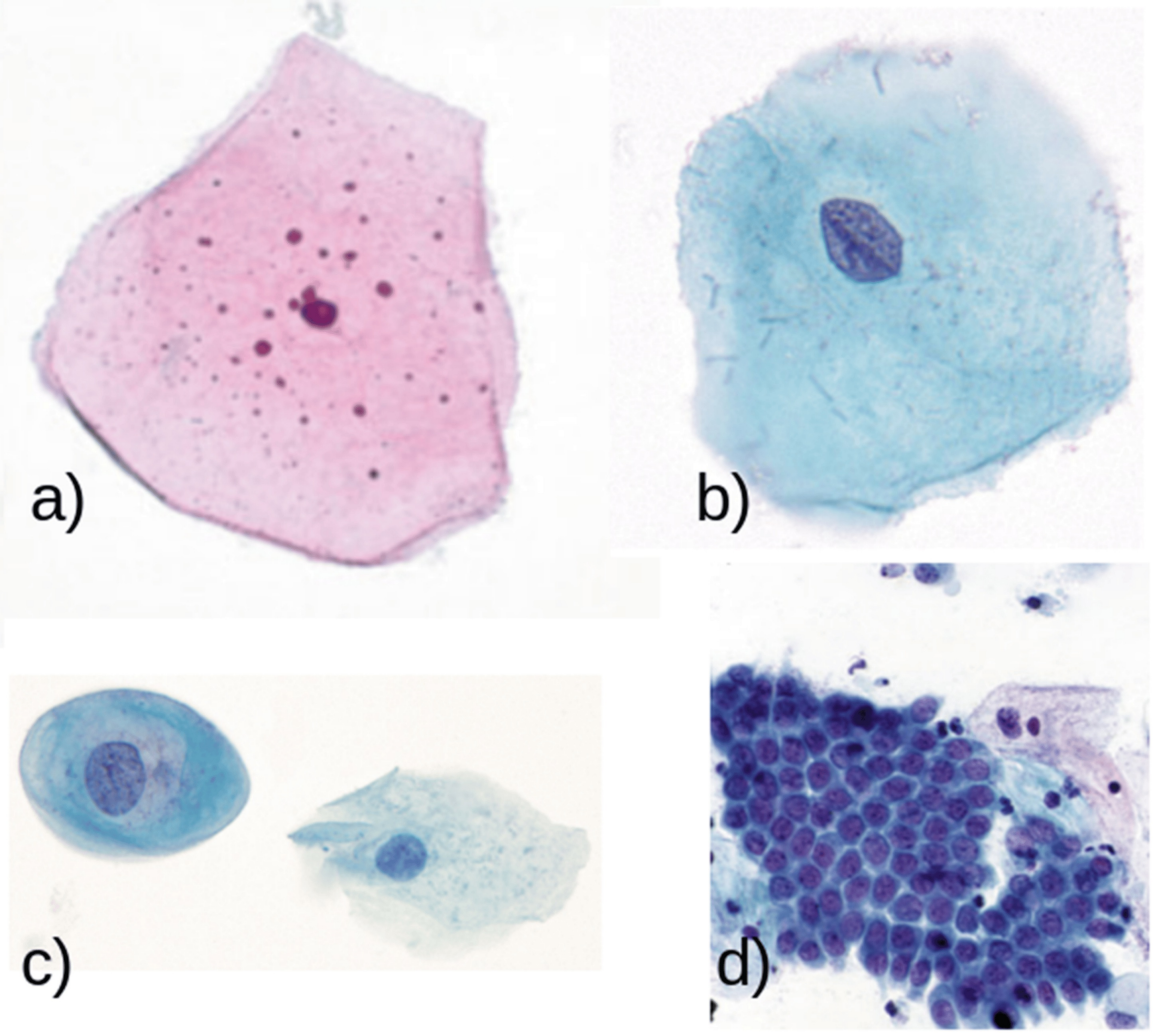
a) Superficial cell, b) intermediate cell, c) parabasal cell, d) endocervical cell.

For instance, the superficial cells in Figure 5a were derived from the outermost layer of the cervical epithelium and are typically visible during the proliferative phase of the menstrual cycle or in the presence of irritation. The nucleus is highly condensed (pyknotic) and has a cross-sectional area of 10 to 15 µm^2^ [32].

Similarly, the intermediate cell (Figure 5b) has a polygonal cytoplasmic profile and the nucleus exhibits finely granular chromatin with a longitudinal groove. The cross-sectional area of the intermediate nucleus is approximately 35 µm^2^ and is generally used as an internal reference for size comparisons [32].

The parabasal cell (Figure 5c) displays typical characteristics with an oval nucleus, fine chromatin, and a cross-sectional area of approximately 50 µm^2^. The endocervical cells (Figure 5d) can also be seen in the typical honeycomb arrangement of benign glandular epithelium, with a cross-sectional area of 50 µm^2^.

However, the primary aim of this work is not to classify each cell but to identify and extract the cells present in a cytology sample, regardless of variations in color, size, staining, or shape, in real time. The ultimate goal is to contribute to the detection of precursor lesions of cervical cancer in the analyzed cells.

**Figure 6:**
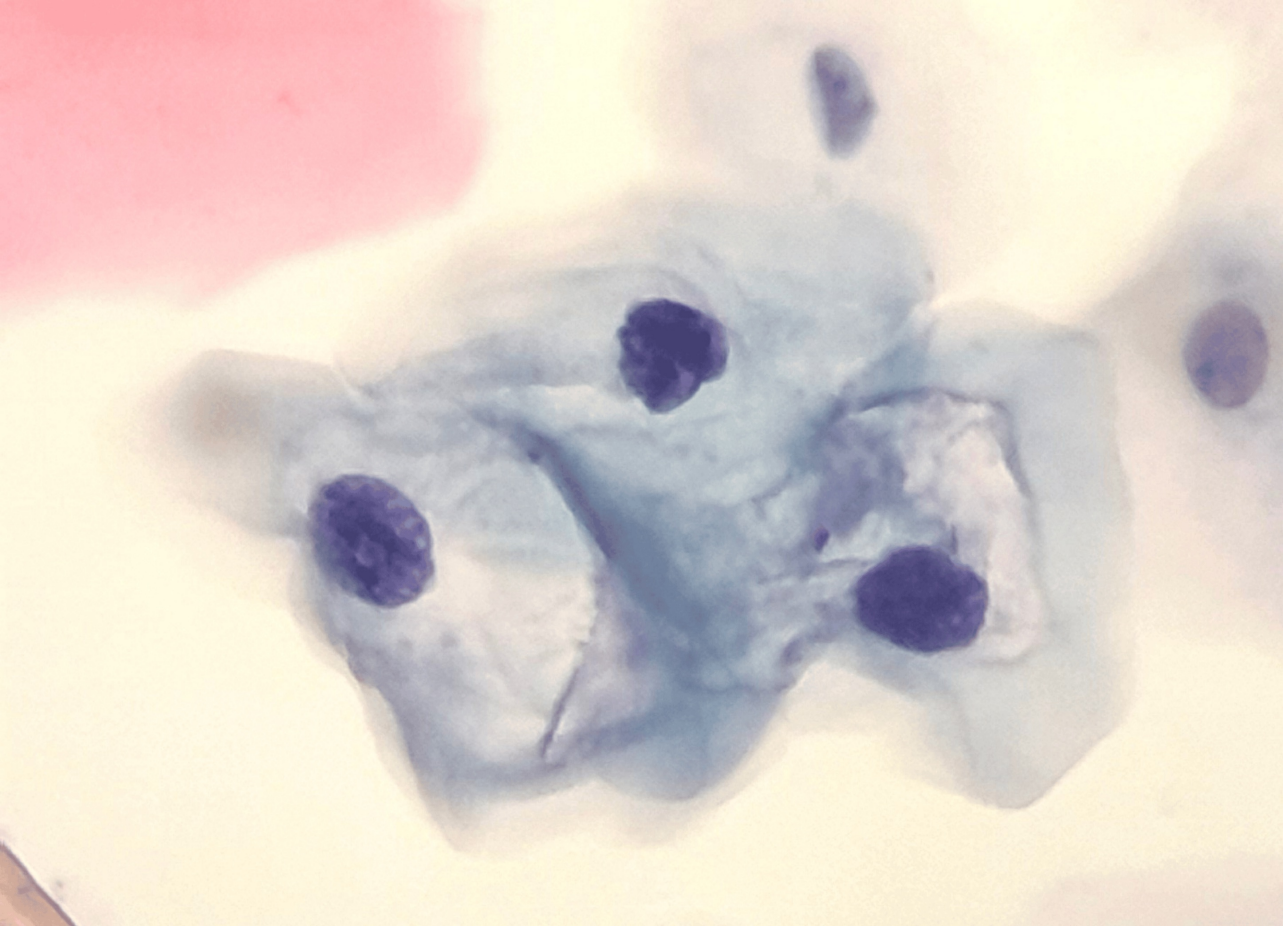
Low-grade cervical cytology lesion.

An example of this is high- and low-grade squamous intraepithelial lesions [33], as illustrated in Figure 6, where a low-grade intraepithelial lesion with typical morphological changes associated with HPV infection is shown, corresponding to a koilocyte.

**Figure 7:**
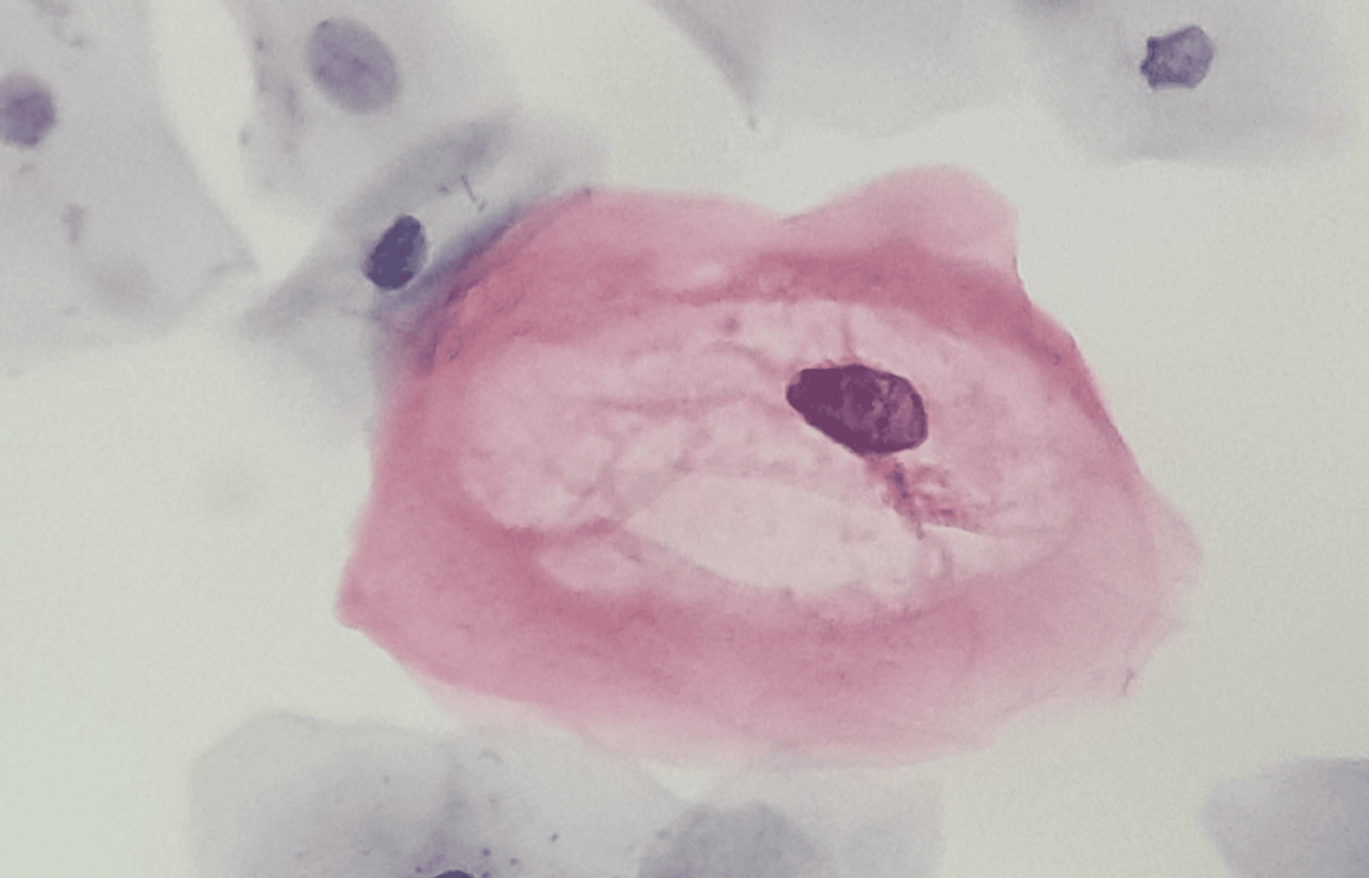
Koilocyte image

A koilocyte is a squamous epithelial cell commonly found in the superficial and intermediate layers, although it can also be observed in parabasal and metaplastic cells [33]. The koilocyte presents typical changes in its nucleus and cytoplasm, losing the usual angular edges of the superficial squamous cell, and its shape tends to become round or ovoid [33]. The cytoplasm shows peripheral condensation, giving it a wire-like appearance; it is opaque, dense, and exhibits a waxy, amphophilic, acidophilic, or bright red/orange color [33], as seen in Figure 7.

Since the koilocyte originates from superficial and intermediate squamous cells, as illustrated in Figure 7, it is crucial to detect all visible cells in the cervical cytology sample, even when their morphology varies. Figure 4 provides an example of the different cell types present in a cytology sample, showing a low-grade squamous intraepithelial lesion alongside squamous cells, paranasal cells, and koilocytes.

Theory and calculation

As previously mentioned, this work proposes using blob detection with the normalized scale LoG algorithm as a segmentation method.

Region Detection: LoG

The LoG operator is one of the most widely used region detectors. The input image *f(x, y)* is convolved with a Gaussian kernel according to the following equation (1).

$g\left( x,y,\sigma \right)=\frac{1 }{2\pi \sigma}exp^{\left( x^{2}+y^{2} \right)/2\sigma}$

where the variable *t* in the equation determines the scale, providing the space-scale representation shown in (2).

$L(x, y, \sigma) = g(x, y, \sigma) &lowast; f(x, y)$ (2) 

Thus, the Laplacian operator is defined by (3)

$\nabla^2L = L_{xx}+ L_{yy}$ (3)

The LoG has a strong positive response for dark regions of size $\sigma$ and a negative one for bright same-size regions. However, its response depends highly on the relationship between the region size and the size of the Gaussian kernel used.

It is necessary to approximate different scales to detect regions of varying sizes automatically. One way to achieve this is by considering the normalized scale Laplacian (4).

$\nabla^2_{norm}L(x,y;\sigma) = t(L_{xx}+ L_{yy})$ (4)

Next, the maxima-minima of the space-scale representation are detected, which are simultaneously local maxima-minima of $\bigtriangledown^{2}_{norm}L$ with respect to both space and scale.

Thus, given a two-dimensional discrete image* (x,y)*, a discrete three-dimensional space-scale volume *L(x,y,*$\sigma$ *) * is formed, and a point is considered a bright region if its value is greater than that of its 24 neighbors (and lower for dark regions).

In this way, interest points $(\hat{x},\hat{y})$ and scales $\hat{\sigma}$ are selected simultaneously, as indicated by equation (5).

$({\hat {x}},{\hat {y}};{\hat {\sigma}})=\operatorname {argmaxminlocal} _{(x,y;\sigma)}(\nabla _{norm}^{2}L(x,y;\sigma))$ (5)

Some basic properties of these regions are that their responses are covariant to translation, rotation, and scale changes in the image domain. Thus, if a space-scale maximum is found at the point *(x_0_, y_0_; $\sigma_0$ )*, resizing the image by a scale factor s will result in a space-scale maximum at * (sx_0_, sy_0_;* s^2^ $\sigma_0$ *) *in the resized image. This property is useful in practice and used for scale selection in other contexts, such as corner detection and object recognition, generally adding robustness to geometric changes.

The methods implemented in the proposed system for cell detection and segmentation were developed using Python and C. For the creation of the cell datasets, 500 slides were provided by the State Public Health Laboratory of Michoacán, with information supplied by specialists in cervical cytology. In addition, the average time cytotechnologists take to analyze and detect malignant cells was considered. This approach focuses exclusively on detecting and analyzing regions of interest (blobs), without employing machine learning or deep learning techniques, as no individual cell classification is performed.

However, a complementary approach using SIFT descriptors previously developed is used in this methodology. The segments extracted from this work enabled the creation of a descriptor knowledge base, facilitating cell tracking and detection. 

## Results

Cell segmentation using blob detection

**Figure 8:**
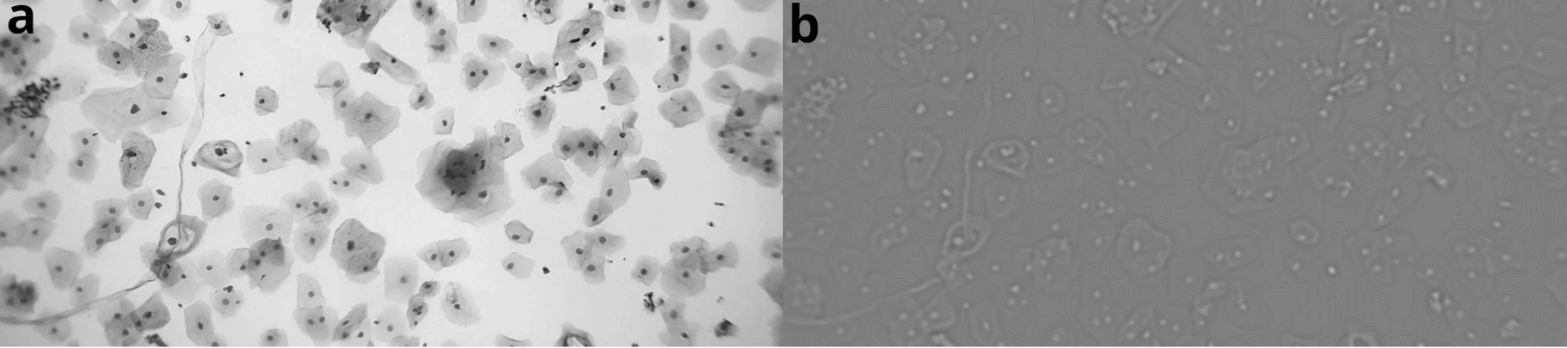
Application of the Laplacian of Gaussian with a fixed σ value, visibly highlighting the cell nuclei: a) original image, b) modified image

More than 15,000 images were extracted from video sweeps of cervical cytology slides, with varying resolutions, the highest being 3840 x 2160 px. The videos were captured using microscopes equipped with 10x and 40x objectives, and an additional 2x zoom was applied using a mobile device, as shown in Figure 3. In Figure 8, the LoG filter highlighted the regions of interest, particularly the nuclear centers of the cells, by adjusting the σ value to enhance detection. However, this process alone is insufficient for identifying regions across different scales, emphasizing the need for complementary techniques to enable multiscale analysis.

To cope with this, the normalized scale LoG is employed, a widely used technique in image processing and computer vision for detecting relevant structures such as edges and feature points across multiple scales. This method combines initial Gaussian smoothing, which reduces noise and small variations in the image, with the Laplacian operator, which accentuates the most significant intensity transitions, such as edges.

**Figure 9:**
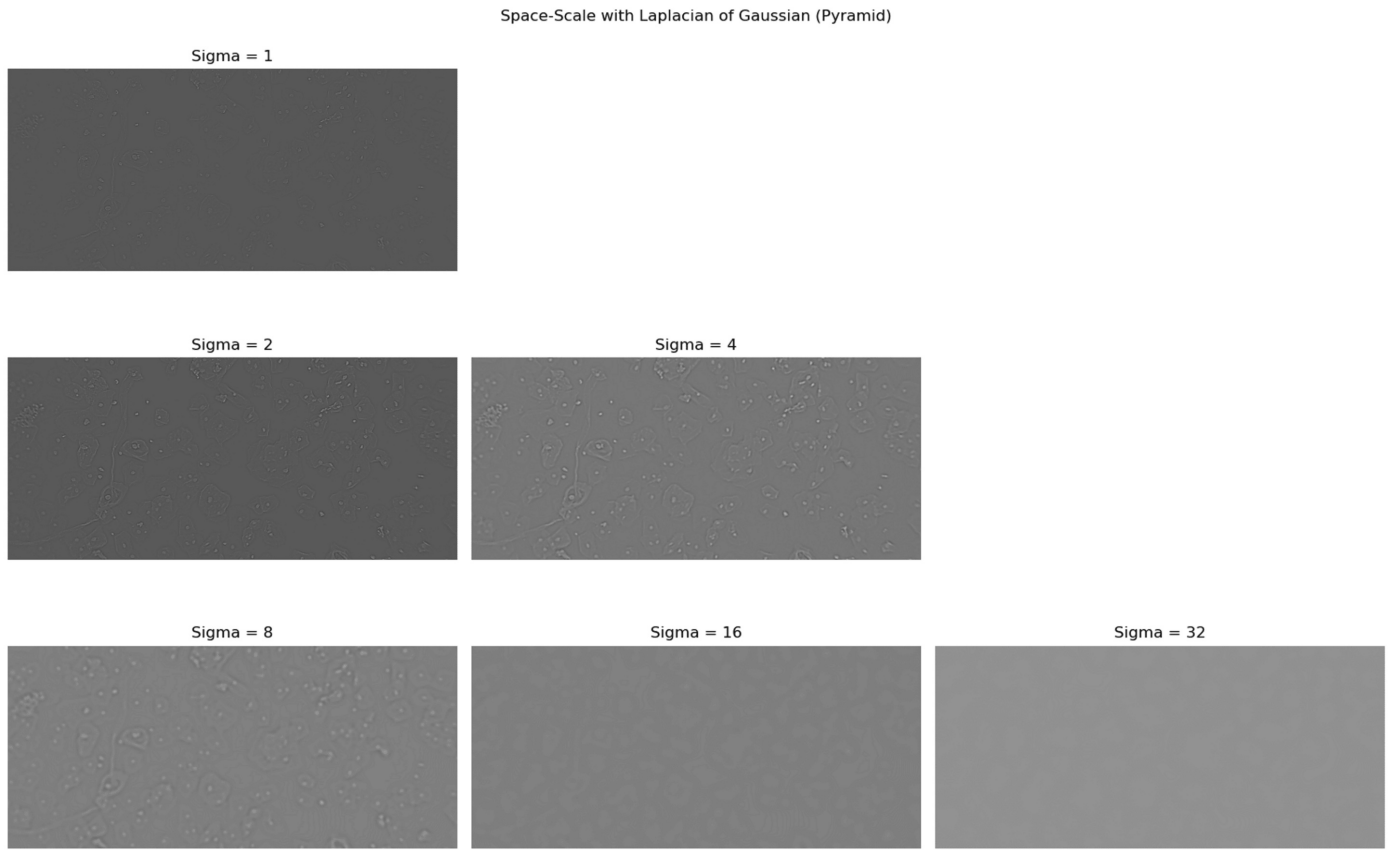
Scale-space pyramid with different sigma values, where the cell nuclei are highlighted with each applied value.

Scale normalization ensures the detected features are consistent across different sizes or resolutions. For this, the Laplacian of the Gaussian operator is multiplied by the square of the scale $\sigma^2$, adjusting the result to make it independent of object size in the image. Thanks to this normalization, the operator can effectively detect the same features, such as the nuclear centers of cells or regions of interest, considering various resolutions and sizes in an image (Figure 9).

Therefore, using the normalized scale LoG allows the analyzed images without uniform staining, specific resolution, or focal distance for region detection.

**Figure 10:**
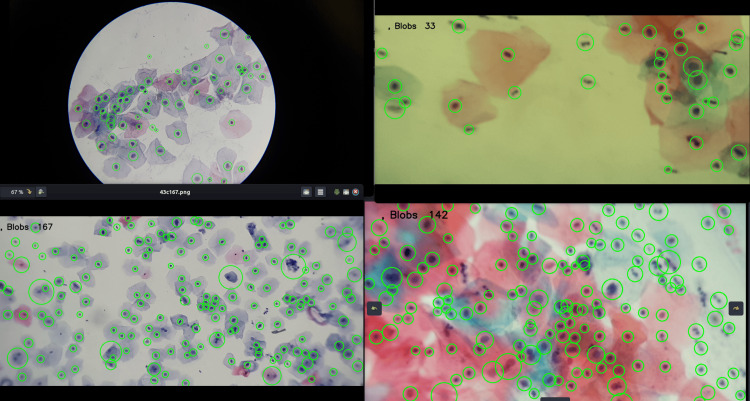
Blob detection in a cervical cytology image with different zoom levels and staining.

Figure 10 presents cervical cytology images with different colors and zoom levels, obtained from slides provided by the State Public Health Laboratory of Michoacán, Mexico. However, when processing these images and applying the LoG, it is possible to correctly identify the blobs, as illustrated in Figure 10.

Although various machine learning and computer vision methods exist, region of interest detection enables the localization of cell nuclei without resorting to exhaustive deep learning processes.

Specific analysis of the nuclei using the LoG facilitates the detection of all types of cells, including those with low- and high-grade lesions, opening the possibility for developing new studies or strategies to identify cellular damage.

**Figure 11:**
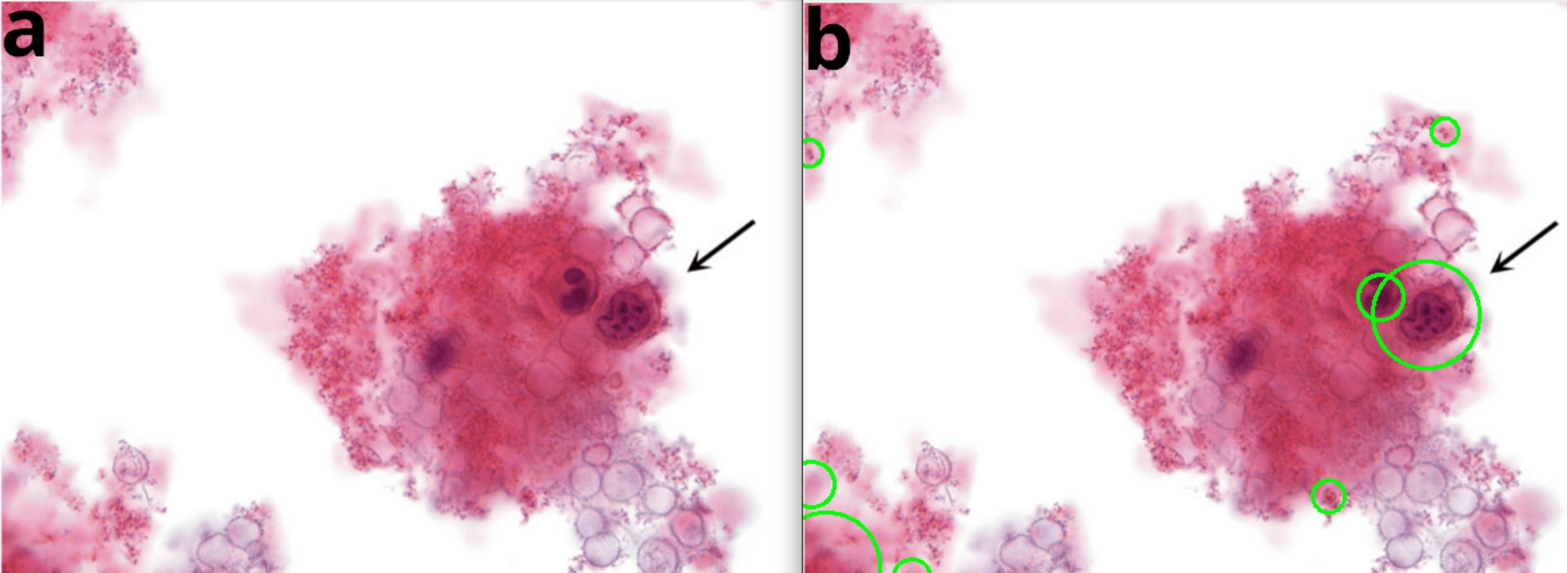
Cervical cytology sample with a bloody appearance, where blood may obstruct the ThinPrep filter. a) Original image, b) processed image. These samples may contain few cells and be technically unsatisfactory. Reference: [32]

These alterations may manifest in the morphology of the cells, as a positive diagnosis typically leads to changes in the size and shape of the nucleus. In addition, the staining and structure of the cytoplasm may be affected. For instance, Figure 11 shows a blood sample in which squamous cell carcinomas are frequently identified.

Since blood can obstruct the ThinPrep filter used in cytology sample collection, these samples may contain a limited number of cells and be technically unsatisfactory. However, even unsatisfactory bloody samples should be examined to detect rare abnormal cells buried in the blood, as indicated by the arrow in Figure 11.

**Figure 12:**
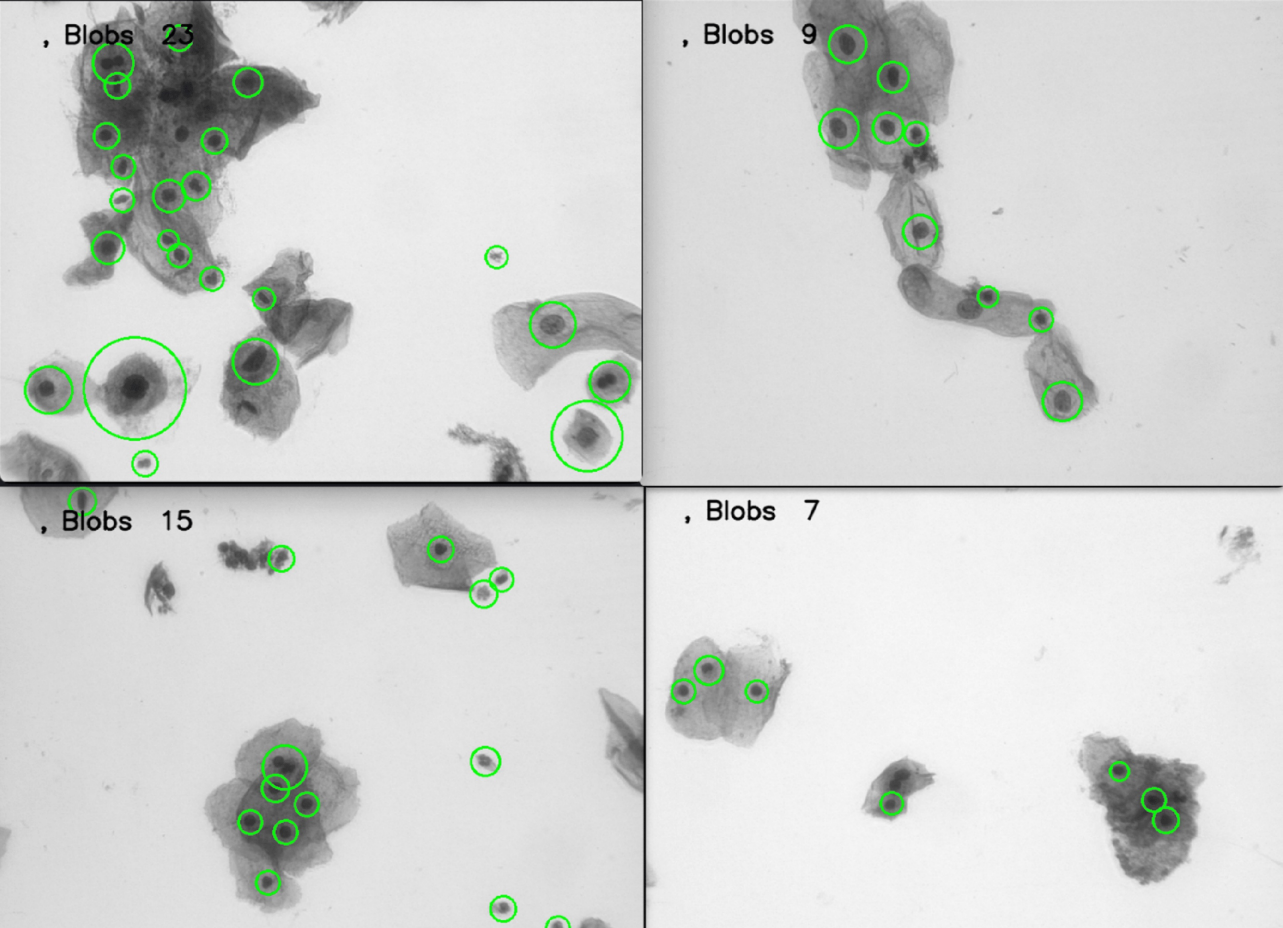
Blob detection in cervical cytology samples from the ISBI 2014-2015 dataset. Reference: [34]

Although a large volume of images was generated from the slides provided by the State Public Health Laboratory of Michoacán, this methodology was also tested with datasets available in the literature, such as those presented in [34], some of which are shown in Figure 12.

In adequately stained images, the process successfully detects 99% of visible cells. However, the proposed methodology aims to detect cells under any condition, as long as they are visible.

**Figure 13:**
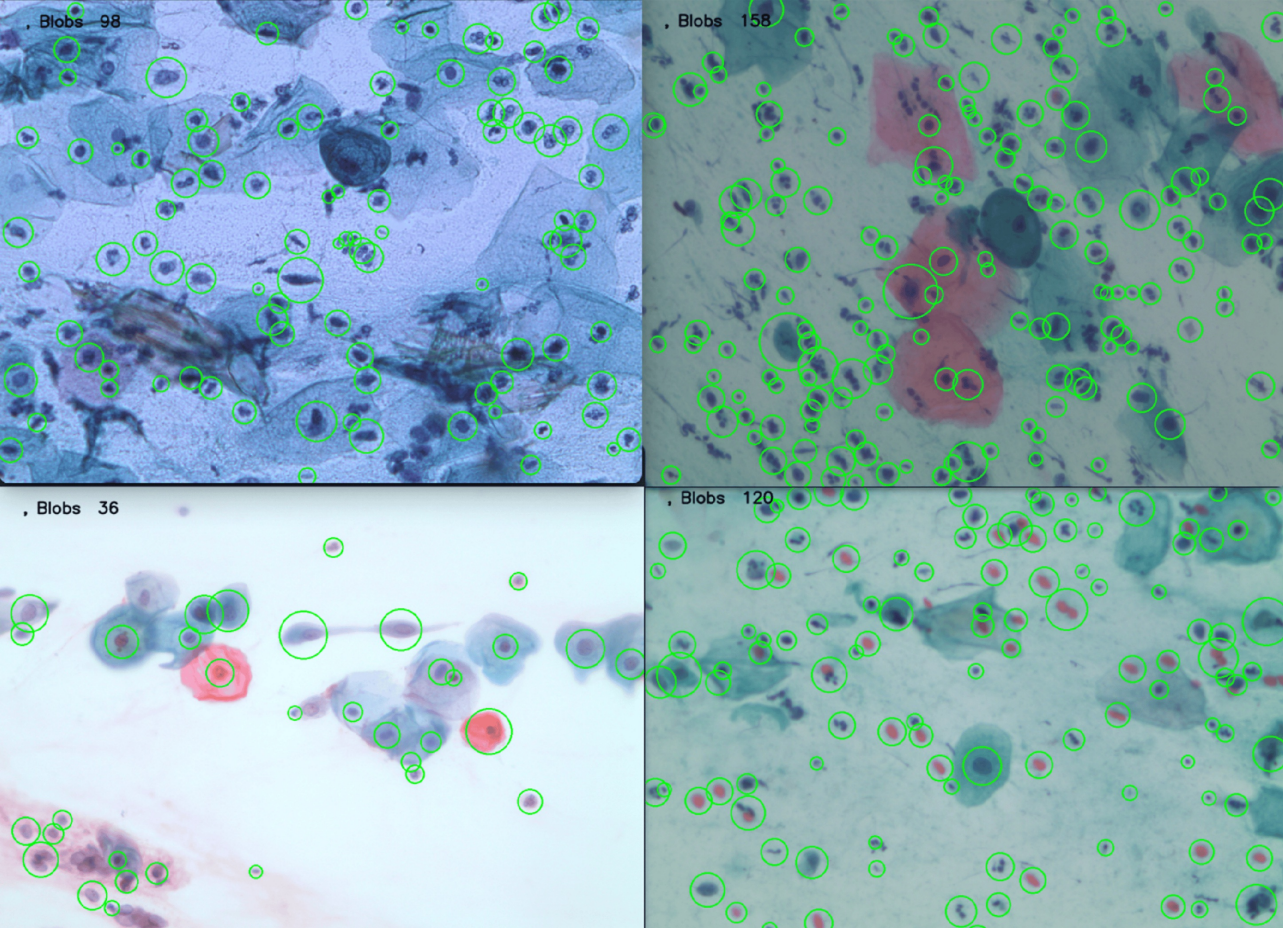
Blob detection in cervical cytology samples from the RepoMedUNM dataset. [35]

In addition, tests were conducted with the dataset available at [35], yielding the results shown in Figure 13.

**Figure 14:**
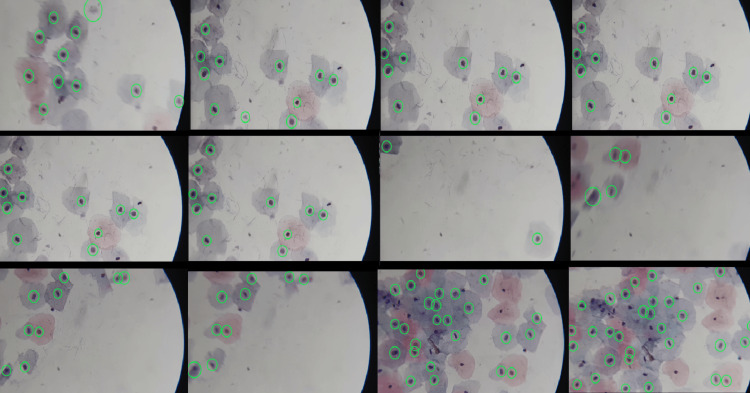
Blob detection in a video sequence (captured using a 1x zoom on the mobile device and a 10x microscope objective).

Figure 14 represents the same procedure, illustrated through a video sequence, blob detection is shown in each video frame, providing a detailed and progressive description of the process.

**Figure 15:**
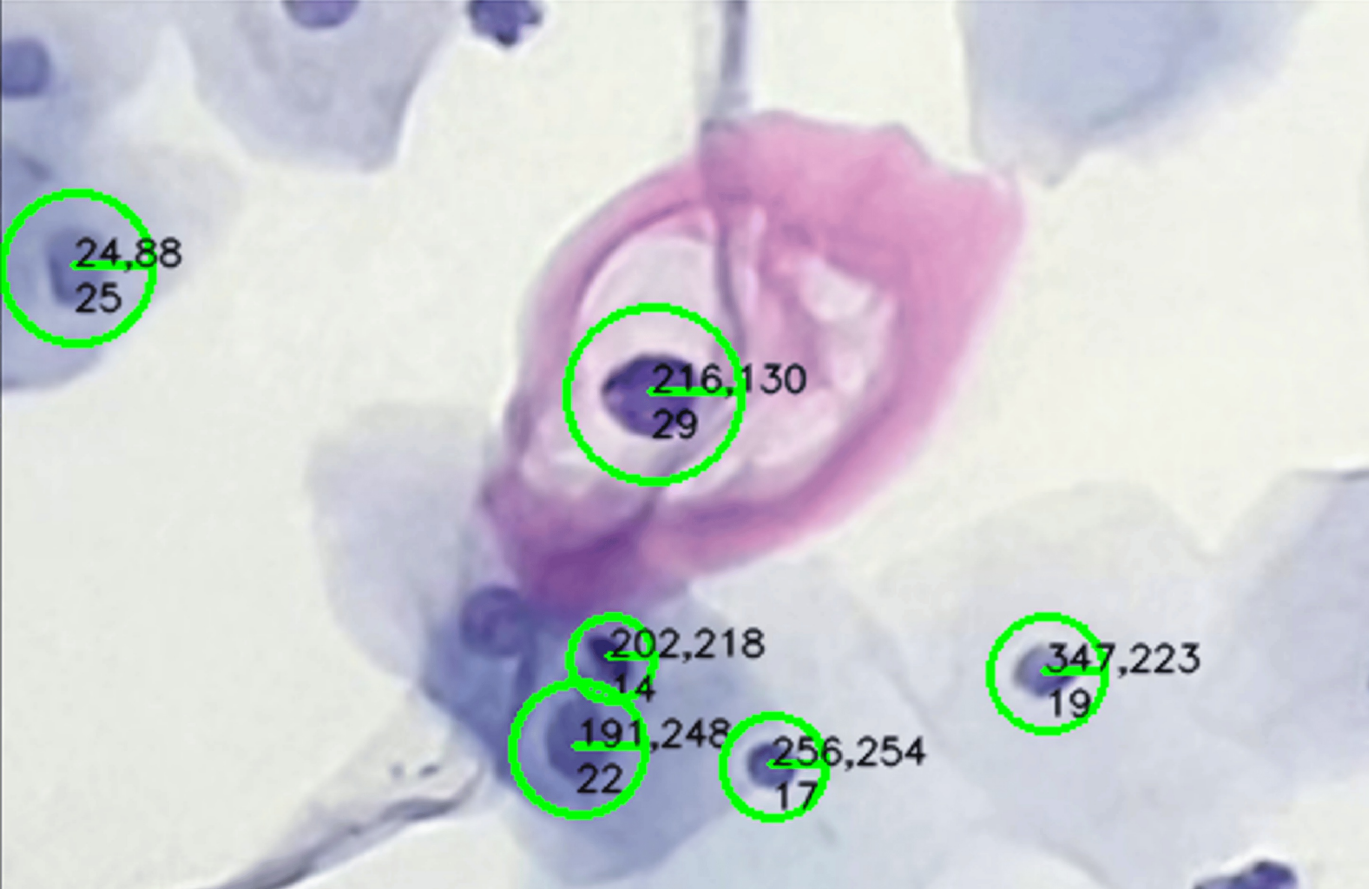
The sample shows a low-grade lesion in cervical cytology. Visible coordinates and radius generated during blob detection.

Therefore, blob detection and generation represent a highly effective strategy for the rapid and precise extraction of the cell nucleus and cytoplasm. During the detection process, it is possible to identify the coordinates and size of each blob, as shown in Figure 15.

Using the LoG, blobs are detected by identifying regions with significant intensity changes in the second derivative. Stable pixels are defined as those that contribute to the maxima or minima in the response of the LoG operator.

In Figure 15, the size of the blob is calculated from these stable pixels within the region, allowing segmentation of the area by focusing on the center and distribution of the blob. The parameter r is linked to the scale of the filter σ and is calculated as $r=\sqrt{2}.\sigma$, ensuring that the detected blobs are consistently represented across varying sizes and regions. These data are essential for the quick and efficient extraction of the sample, as the segment can be delimited based on the parameters represented in the matrix shown in Equation (6), where the relevant values correspond to corners generated through the size and center of the blob.

\begin{equation} \begin{pmatrix} (x-r, y-r) & 0 & (x+r, y-r)\\ 0 & (x ,y) & 0 \\ (x-r, y+r) & 0 & (x+r, y+r) \end{pmatrix} \label{mate} \end{equation} (6)

**Figure 16:**
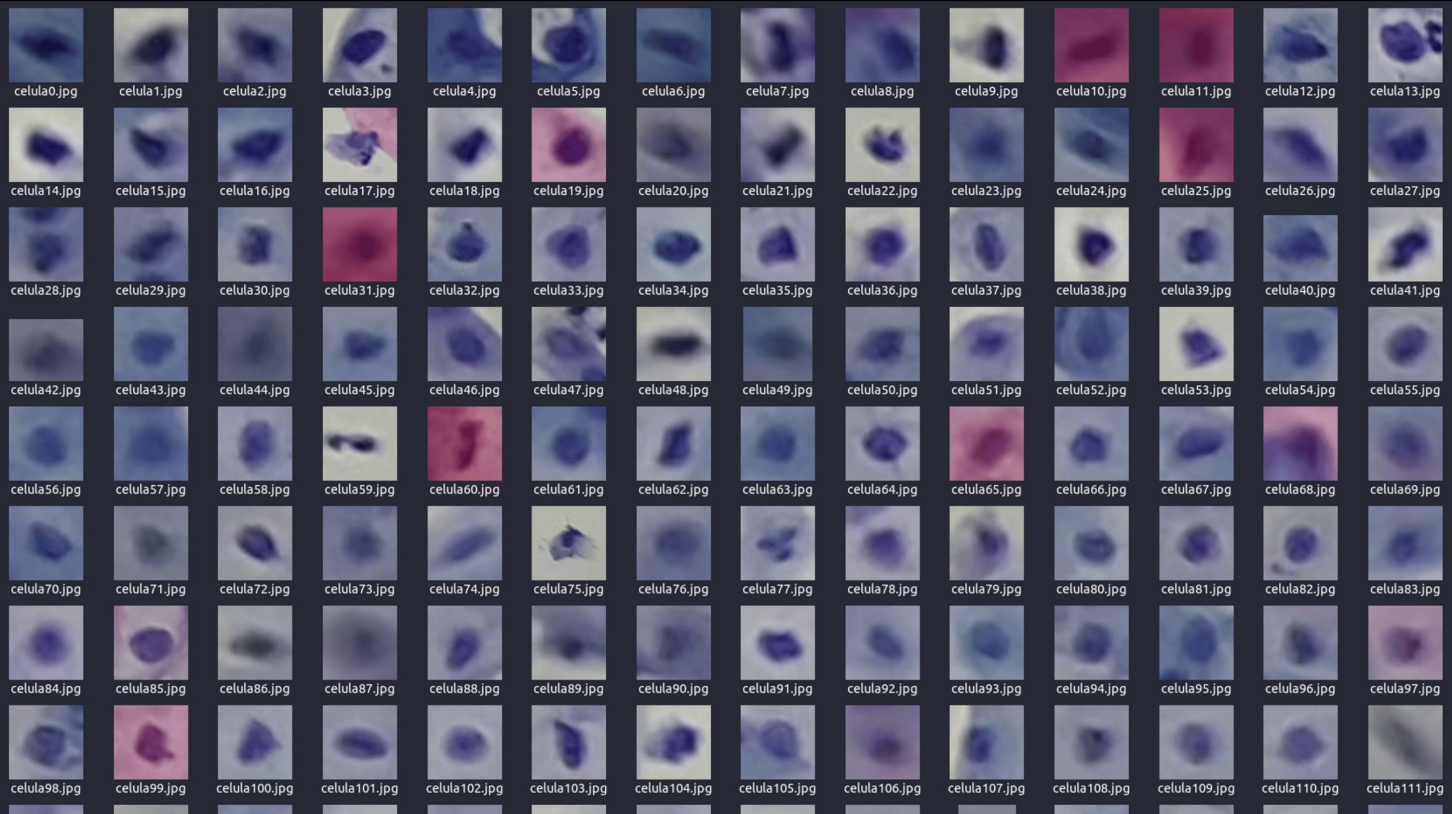
Segmented cells obtained from the detected coordinates and the established blob radius

Thus, the value of r can be adjusted, increasing or decreasing, to extract slightly larger or more precise regions of the cellular area. This adjustment allows greater flexibility in delineating areas of interest, depending on the needs of the analysis. This fast-cutting and extraction process can be applied to video sequences and static images, as illustrated in Figure 16, where the cell nuclei and part of the cytoplasm are observed.

This automated method eliminates the need to crop manually each visible cell in a cervical cytology sample, significantly optimizing the data extraction process. The primary goal of this technique is to accelerate the analysis and improve efficiency, especially in a context where cytology samples often present variability and lack exact reproducibility. Furthermore, this procedure allows the extraction of key information from the cell nucleus, which can be critical for identifying abnormalities and enhancing the accuracy and speed of diagnosis.

Final results

All implementations and validation tests were performed on personal computers equipped with Intel Core i7 and i5 processors and a maximum of 8 GB of RAM. This approach was deliberately chosen to demonstrate the feasibility of the proposed methodology in resource-constrained environments, such as healthcare centers in underserved communities with limited computational infrastructure. Additionally, the entire process was validated by the State Public Health Laboratory of Michoacán, ensuring the reliability of the results in applied and clinical contexts. 

Once the values derived from the application of the LoG algorithm demonstrated consistent success across all evaluated cases, the performance of the method was assessed using quantitative metrics. Widely recognized metrics, such as precision, accuracy, sensitivity, and the F1 score, were employed to evaluate the algorithm’s performance, particularly in cell detection and counting tasks. These metrics provided a comprehensive evaluation of the method, ensuring its validity for real-world cytological analysis. 

Moreover, a detailed comparison of results was conducted across various experimental conditions to confirm the robustness of the proposed approach. This thorough analysis highlights the algorithm’s adaptability and effectiveness, even under diverse testing scenarios, positioning it as a reliable alternative for cytological applications. 

Similarly, some studies focus on cell segmentation using blobs or machine learning algorithms. This analysis is commonly applied to datasets such as ALL-IDB, ISBI, and CX22, among others. One example is presented in [20], which uses the ISBI dataset and incorporates real-world private data. In the first case, the precision was 0.978 with a sensitivity of 0.93; however, when applying real-world data, the precision dropped to 0.770, with a sensitivity of 0.886. Similarly, [14] applies blob detection and a deep learning method using the ALL-IDB dataset, involving 33 images, achieving a precision of 1 for the first two sets and 0.997 for white blood cell detection.

In the context of this research, the analyzed data comes from the State Public Health Laboratory of Michoacán. We received a set of approximately 500 slides, each characterized by a wide range of attributes. The provided slides include Pap smear samples, ranging from those considered adequate and processed with proper staining to others showing clear defects, either due to incorrect execution or a scarcity of cells present.

This intentional selection of diverse slides aims to represent a broad range of conditions and scenarios, ensuring the robustness and representativeness of the data. The inclusion of samples varying from adequacy to extreme deficiency is supported by the knowledge and specialized criteria of the laboratory staff.

This approach aims to improve the quality and relevance of the study’s results by using a dataset that faithfully reflects the inherent variability in laboratory practices and the conditions of the analyzed samples.

Similarly, promising results were obtained when applying the method to blocks of 100 images. However, the process has the potential to be implemented in real-time video sequences, achieving a precision of 96.5%, accuracy of 99.2%, recall of 99.2%, and an F1-score of 97.83, as detailed in Tables 1-2. It is important to mention that the accuracy, precision, recall, and F-measure values in our method were obtained by comparison with the knowledge base mentioned before, which was validated by comparison with the manual cell detection performed by medical specialists from the State Public Health Laboratory.

**Table 1:** Evaluation of cell detection performance compared to leading techniques in the field. The proposed method presents the results achieved in the initial 100 frames analyzed.

	Hady Ahmady [20]	Di Ruberto [36]	Loddo [37]	Diruberto [14]	Our method
N° images	-	-	33	33	100
Accuracy	-	-	-	98.0%	96.5%
Precision	97.8%	89.0%	89.0%	98.7%	96 %
Recall	93.3%	98.0%	98.0%	99.3%	99.2%
F-measure	95.5%	93.0%	93.0%	98.9%	97.8%

Table 1 presents the comparison of the results between studies employing similar processes for cell detection. However, this work does not address the specific classification of lesions or precancerous cells so this comparison is not included. Since no machine learning or deep learning algorithms were used, the main focus of this study is on the general detection and extraction of cells, rather than on the particular classification of precancerous cells.

**Figure 17:**
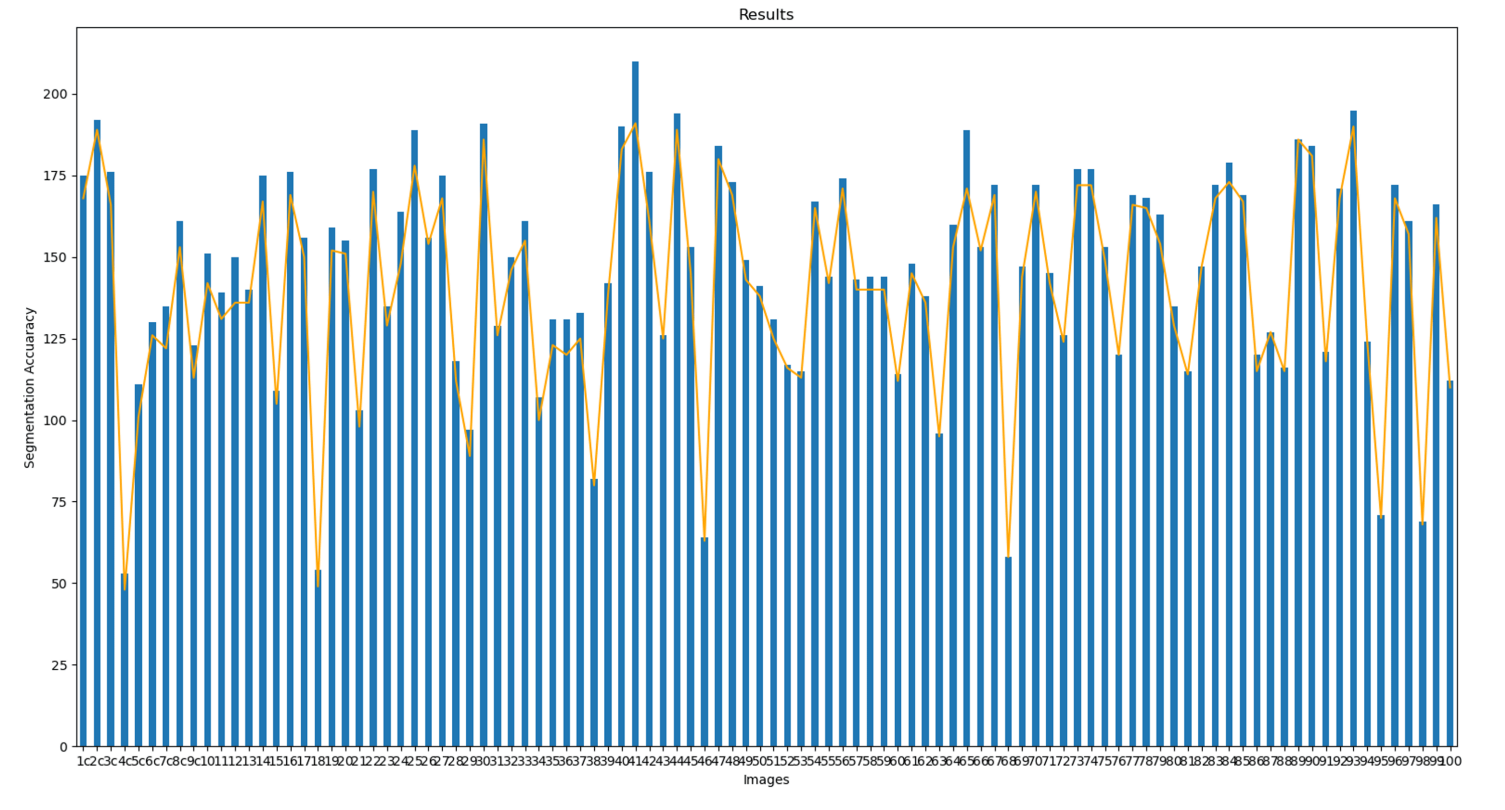
Performance evaluation of the proposed method for cell segmentation. The results from analyzing the initial 100 frames demonstrate the efficacy of the implemented technique

In addition, an analysis was performed by counting the number of segments in each blob based on the radius and coordinates of each element as well as the total number of cells in each frame extracted from the video. In Figure 17, the bars show the total number of cells detected in each frame and the orange line represents the total number of segments in each image.

The extraction accuracy depends directly on the correct detection of cells. As observed, most undetected cells are due to unstable blobs; however, these cases are minimal. This demonstrates that cell extraction is feasible across the entire cytological field without needing ideal cytology concerning staining or specific zoom level, enabling rapid and efficient data extraction. It is worth noting that this study used samples provided by the State Public Health Laboratory of Michoacán, which encompass a variety of staining levels and include both negative and positive cases, offering a comprehensive analysis.

Furthermore, the importance of the experience and skill of specialists when examining slides under the microscope is emphasized, including the speed with which they identify precancerous or fully infected cells. This experience justifies the focus of this work on achieving real-time detection and segmentation.

## Discussion

Blob detection is presented as an efficient alternative to machine learning-based approaches for cell segmentation and detection, particularly in cytology. This method allows for the localization of features such as cell nuclei without requiring extensive training or large amounts of labeled data, making it a more practical option in contexts where data collection is limited.

In addition, blob detection is less computationally expensive, as it does not demand specialized hardware like GPUs, facilitating real-time implementation on devices with limited resources, such as mobile phones connected to microscopes.

Unlike machine learning models, which can be influenced by biases in training data, the blob-based approach is more interpretable, straightforward, and reliable.

This method is also robust to variations in images, such as changes in staining or resolution, avoiding the need for constant adjustments or retraining, which is common in machine learning systems. Due to these fundamental differences in methodology, data, and hardware requirements, it is not possible to directly compare the results obtained with blob detection to those from studies using machine learning or deep learning, as the objectives and approaches are distinct. While machine learning focuses on classification following complex training, the blob method aims for the immediate and general detection of cells without prior learning, highlighting a crucial difference between the two approaches and their applications.

Although most of this study focused on slides provided by the State Public Health Laboratory of Michoacán, datasets used by various studies described in the state of the art were also analyzed. Therefore, Table 2 presents a comparison between the dataset provided by the laboratory and other datasets found in the literature. Since this is a detection and segmentation process, the same metrics and comparisons were applied.

**Table 2:** Performance evaluation of cell detection for various literature datasets compared to cytologies provided by the State Public Health Laboratory of Michoacán (SPHLM).

	N° images	Accuracy	Precision	Recall	F-measure
ISBI2015 [34]	16	98 %	100%	98%	98.99%
SIPakMeD [38]	16	93.33%	100%	93.33%	96.55%
CERVIX93 [39]	16	97.22%	100%	97.22%	98.59%
BTTFA [40]	16	97.5%	100%	97.5%	98.73%
MendeleyLBC [41]	16	96.67%	100%	96.67%	98.31%
CCEDD [42]	16	97.5%	100%	97.5%	98.73%
SPHLM	16	96.67%	100%	96.67%	98.31%

A set of only 16 images was used, as some datasets, such as ISBI2015, contained only that number of cervical cytology images. Despite this limitation, the results obtained are promising, as the images show considerable variability in staining. In some cases, images with more uniform staining demonstrated better performance concerning cell detection and segmentation, suggesting that the algorithm responds favorably under conditions of staining homogeneity.

**Figure 18:**
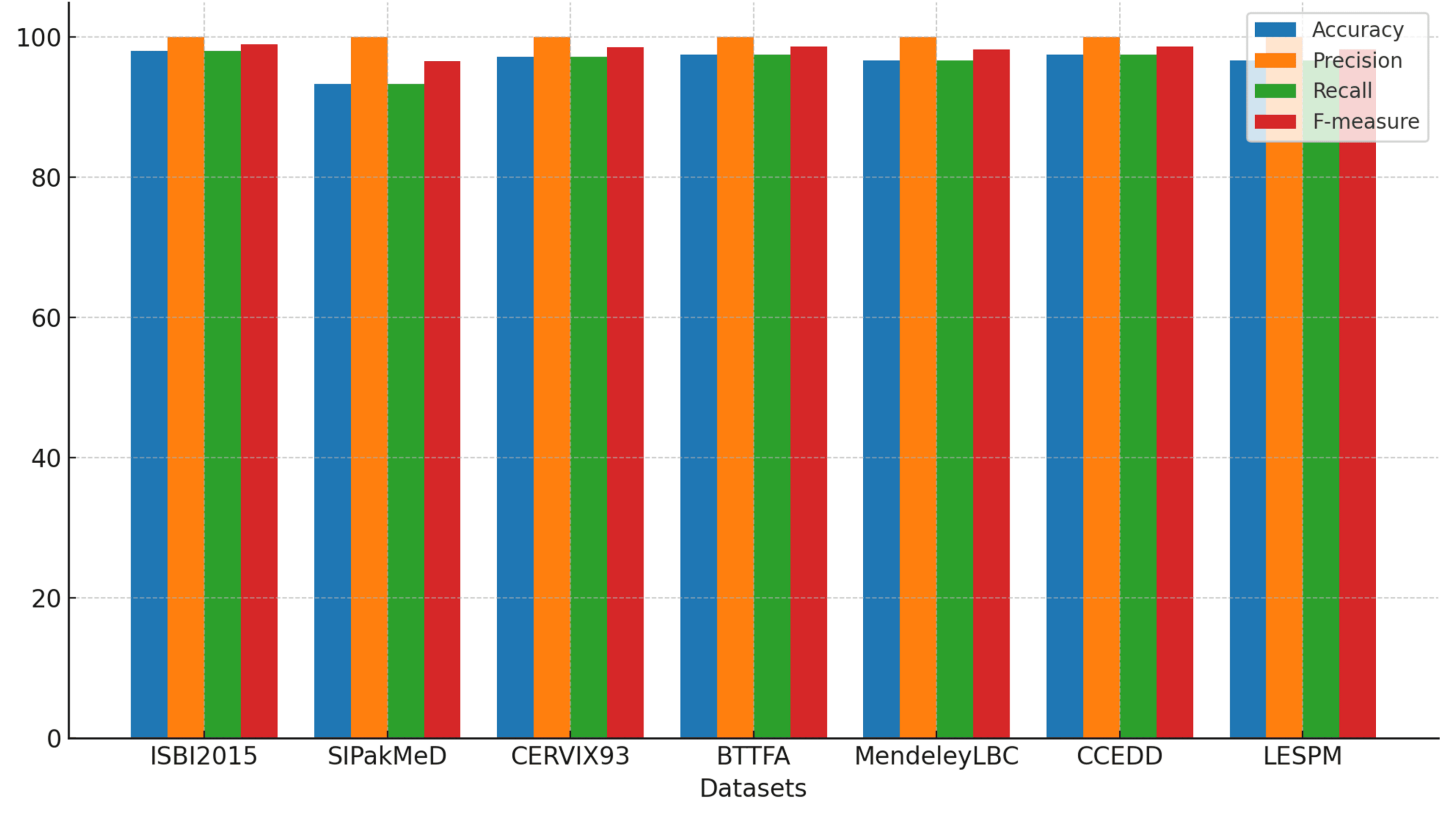
Performance evaluation of the proposed method for cell detection and segmentation using literature datasets, probing the efficacy and robustness of the implemented technique.

However, it is important to highlight that the algorithm adapted robustly even to images with more irregular staining, maintaining high performance in terms of precision and segmentation. This reinforces its potential for application in clinical scenarios with diverse image quality conditions. Figure 18 shows the graphical representation of the results.

**Figure 19:**
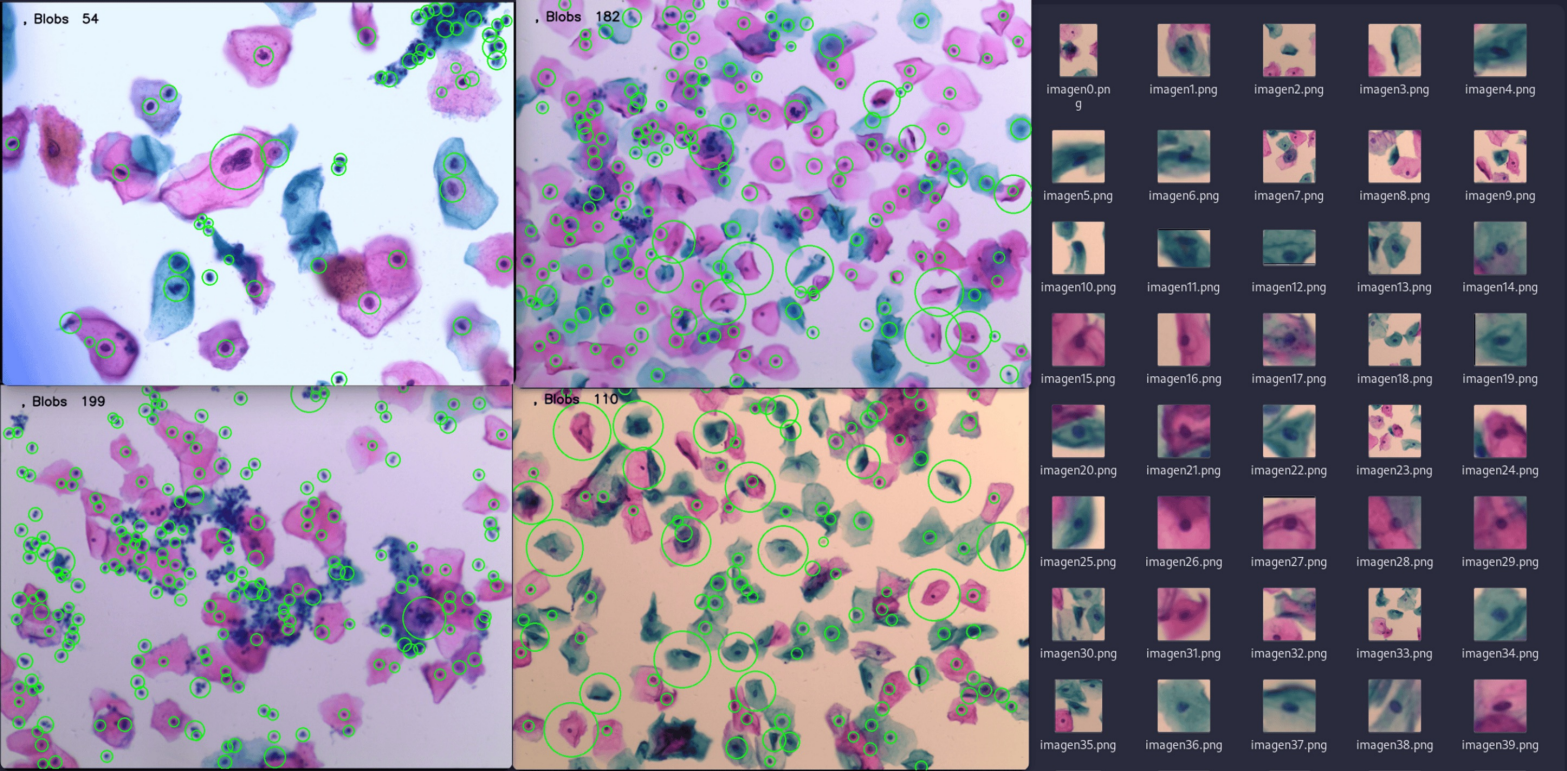
Performance evaluation of the proposed method for cell detection and segmentation using literature datasets. The results obtained from analyzing the first 16 frames confirm the efficacy and robustness of the implemented technique.

Figure 19 shows the cell detection and extraction performance across different cytologies from other used datasets. These results reflect the consistency of the algorithm when handling different types of images, including variations in staining and sample quality. Despite the differences between datasets, the algorithm maintains a high level of precision and effectiveness in cell segmentation, highlighting its robustness and versatility under various experimental conditions.

Limitations of the study

The combination of the techniques presented in this article provides a valuable tool to improve the efficiency and accuracy of cell detection and extraction in cervical cytology. However, it is necessary to evaluate in a subsequent study the effects of variability in the samples, particularly given the uniqueness of each sample, as well as the adaptation to real clinical conditions.

## Conclusions

In this work, the use of the LoG, along with a proposed formula based on the coordinates and size of pixels exhibiting stable coloration, was implemented to extract regions of interest in cytological images. While this approach does not specifically classify or extract malignant neoplasms, its main strength lies in its adaptability and robustness to variations in cytological images generated from cervical cytology slides. This allows it to extract all types of cells or cellular components present in any cytology. This approach offers an adaptable tool for cytological image analysis that could be further refined to cover a broader range of applications, including the automatic detection of precancerous lesions.

It is important to highlight that this methodology stems from findings in the literature, which indicate that slides generated through Pap smear techniques lack reproducibility, leading us to avoid using machine learning or deep learning algorithms due to their long training processes, developing an approach that is adaptable enough to function in real-time. For this reason, computer vision algorithms that could contribute to a more efficient and flexible detection in all possible cases were used. Despite this, the results are promising, and further work can enhance the cervical cancer detection process, providing a reliable tool to analyze cervical cytologies.
